# Species interactions constrain adaptation and preserve ecological stability in an experimental microbial community

**DOI:** 10.1038/s41396-022-01191-1

**Published:** 2022-01-22

**Authors:** Jake N. Barber, Luke C. Nicholson, Laura C. Woods, Louise M. Judd, Aysha L. Sezmis, Jane Hawkey, Kathryn E. Holt, Michael J. McDonald

**Affiliations:** 1 School of Biological Sciences, Monash University: Clayton, Wellington Road, Clayton, VIC 3000 Australia; 2 Department of Infectious Diseases, Central Clinical School, Monash University, Melbourne, VIC 3004 Australia; 3 Department of Infection Biology, Faculty of Infectious and Tropical Diseases, London School of Hygiene & Tropical Medicine, London, WC1E 7HT UK

**Keywords:** Evolution, Microbial ecology

## Abstract

Species loss within a microbial community can increase resource availability and spur adaptive evolution. Environmental shifts that cause species loss or fluctuations in community composition are expected to become more common, so it is important to understand the evolutionary forces that shape the stability and function of the emergent community. Here we study experimental cultures of a simple, ecologically stable community of *Saccharomyces cerevisiae* and *Lactobacillus plantarum*, in order to understand how the presence or absence of a species impacts coexistence over evolutionary timescales. We found that evolution in coculture led to drastically altered evolutionary outcomes for *L. plantarum*, but not *S. cerevisiae*. Both monoculture- and co-culture-evolved *L. plantarum* evolved dozens of mutations over 925 generations of evolution, but only *L. plantarum* that had evolved in isolation from *S. cerevisiae* lost the capacity to coexist with *S. cerevisiae*. We find that the evolutionary loss of ecological stability corresponds with fitness differences between monoculture-evolved *L. plantarum* and *S. cerevisiae* and genetic changes that repeatedly evolve across the replicate populations of *L. plantarum*. This work shows how coevolution within a community can prevent destabilising evolution in individual species, thereby preserving ecological diversity and stability, despite rapid adaptation.

## Introduction

Microbial communities are bound by a range of competitive and cooperative interactions [1], and a growing number of experimental studies show that changes in species composition can drastically alter the course of evolution for those species that remain [2–13]. Incorporating multiple species into experimental, multigenerational cultures has revealed a range of outcomes, including accelerated molecular and fitness evolution [14, 15], decelerated fitness evolution [14], more efficient use of resources [5, 16–18], and community stabilisation [7, 8, 17]. These results highlight that, in addition to the environment, the identity and role of a species in the community are crucial for determining the outcome of evolution. For example, increasing the number of distinct types of phage—viral parasites of bacteria—that are encountered by a co-cultured host increases the rate of evolution of that host [19]. On the other hand, if a single phage is co-cultured with multiple distinct host bacteria, the rate of phage adaptation to any individual host is slowed [20]. Another result from studies of laboratory bacterial communities is that the opportunities for adaptation diminish when there are more species in the co-culture [21] and that reducing the number of species in a community increases the opportunity for adaptation [16, 22, 23].

Despite this progress, we still know little about the selective forces and genetic changes that underlie the evolution of microbial interactions [10]. In particular, an outstanding question is how the evolution of one species in isolation from the community impacts interspecies interactions, especially for stably coexisting species. Within ecosystems, coexistence is maintained by equalising mechanisms that reduce fitness differences [24**–**26], or stabilising mechanisms that reduce niche overlap, such as character displacement or niche differentiation [24, 27, 28]. In an ecosystem where most resources are utilised, it is not surprising that the local extinction of one species, such as a predator [29, 30], parasite or even an indirect competitor for a resource [31], provides an opportunity for another local species to exploit the newly available resources. The expansion of niche range and population size for one species concomitant with the loss of another species from the community is known as ecological release [32]. Whilst this process is clear for species under intense negative interactions in their original communities, it is unclear if species with positive interactions, such as cross-feeding strains, would also undergo changes in niche requirements. Coexistence theory predicts that if two species compete for the same resources, then one species is likely to drive the other extinct [33, 34]. Therefore, in the absence of stabilising and equalising mechanisms that promote coexistence [34], species that undergo ecological release are likely to increase competition when reintroduced into a community setting.

To address how periods of species loss affect community stability on evolutionary timescales, we establish a simple, ecologically stable laboratory community of two species, the eukaryote *Saccharomyces cerevisiae* and a newly isolated strain of the bacterium *Lactobacillus plantarum*. Both species are commonly recovered from fermented food and beverages [35], and laboratory studies suggest that *L. plantarum* benefits from amino acids secreted by *S. cerevisiae* [36]. Although *S. cerevisiae* is a well-described model organism commonly used for evolution experiments, *L. plantarum* has fewer resources available for genomic and genetic characterisation. In this study, we passage both species in monoculture and co-culture for 925 generations. We measure the fitness of all the evolved and coevolved populations and carry out whole-population sequencing to determine the genetic basis of adaptation to monoculture and co-culture. We then reintroduce monoculture-evolved isolates into co-culture to examine how evolution in isolation alters community stability. Finally, we investigate the relationship between fitness differences and niche overlap in our ancestral and evolved strain pairings to identify a mechanism for interactions between *L. plantarum* and *S. cerevisiae*.

## Methods

### Strains

The *S. cerevisiae* strain used to find the evolution experiment was a haploid, non-recombining derivative of YJM978 (*Mata, ho::HygMX, ura3::KanMX*) [37, 38]. The *L. plantarum* strain used in this study is a novel strain, LPKH, isolated from a sourdough bread culture.

### Growth media

Evolution experiments and all growth experiments were carried out in CSM, which is comprised of Complete Supplement Mixture (CSM, Sunrise Science), 20 g/L glucose, 6.7 g/L nitrogen base, and here was supplemented with tryptophan (0.05 mg/mL) and uracil (0.02 mg/mL). Modifications of this media for specific assays are described below.

### Growth assays

The performance of ancestral strains was assayed by measuring OD_600_ and cell density after 48 hours of growth in either fresh growth media, or “spent” growth media. To generate spent medium for growth assays with *L. plantarum*, single clones of ancestral *S. cerevisiae* YJM978 were grown to saturation in 3 mL of CSM at 28^o^C over 48 hours (*n* = 3), the cells were removed by centrifugation and filtration using a syringe filter with a pore size of 0.2 μm. To generate spent medium for the growth of *S. cerevisiae*, we cultured clones of ancestral *L. plantarum* in separate wells in a 96-well plate, containing 132 μL of fresh media overlaid with a foil seal (*n* = 72). Cells were removed from overnight cultures via centrifugation (10,000 rpm for 2 min) and the supernatant retained. Cells were washed in phosphate-buffered saline via centrifugation before being resuspended in 3 mL of phosphate-buffered saline. The supernatant was filter-sterilised through a syringe filter with a pore size of 0.2 μm. We compared the growth of both species in both spent and fresh medium. To do this, filtered spent medium was added to a 96-well plate, with 128 μL per well, as well as separate wells for fresh media. About 4 μL of resuspended cells were then added to spent medium and fresh media and grown for 48 hours under experimental conditions (*n* = 64). To obtain time-course measurements, replicates were destructively sampled every three hours to measure optical density (*n* = 4 at each timepoint). After 48 hours, the final optical density was measured, and maximum growth rate was calculated over three time points.

### Glucose utilisation assay

Glucose utilisation in our *L. plantarum*- and *S. cerevisiae* evolved strains was measured using a colorimetric enzyme-based Glucose Assay Kit (Sigma-Aldrich, catalogue number MAK263). Single colonies of ancestral (*n* = 4), monoculture (*n* = 4), and co-culture (*n* = 4) strains of both species were grown to saturation separately in 132 μL of CSM in a foil-sealed 96-well plate for 48 hours. After 48 hours, 10 μL of media was extracted from each well and diluted 100-fold in PBS to obtain measurements within the glucose standard-curve measurements. Measured samples were blanked against a glucose standard containing 0 ng/μL of glucose, then compared against a standard curve to estimate that the remaining amount of glucose is spent media of *L. plantarum* or *S. cerevisiae* strains.

### Evolution experiment

To establish the evolution experiment, single clones of *L. plantarum* and *S. cerevisiae* were grown to saturation in Complete Supplement Mixture (CSM) modified with additional amino acids tryptophan and uracil. Co-culture populations were mixed at an initial ratio of 1:1 *L. plantarum*:*S. cerevisiae* and diluted 32-fold into 132 μL of supplemented CSM media, with 96 replicate co-culture populations distributed across a single 96-well plate. The 48-replicate monoculture treatment populations for each species were founded by the same procedure, but with *S. cerevisiae* and *L. plantarum* kept separate. We took a number of measures to reduce the chance of contamination. Co-culture and monoculture populations were propagated on separate 96-well plates and all dilutions were performed using the Hamilton96 liquid Handler with the CO-RE Probe Head, encased in a HEPA-filtered chamber. In addition, all cultures were foil-sealed before incubation, without shaking, at 28 ^o^C. Transfers of cultures into fresh media occurred every 2 days in a 32-fold dilution (4 μL into 128 μL), resulting in 5 generations per transfer. After every 50 generations, populations were mixed with 50 μL of 75% glycerol and archived at −80 ^o^C. The cultures were propagated for around 925 generations of growth and dilution at 28 ^o^C, under non-shaking and oxygen limiting conditions. Six populations from each condition were chosen to track colony forming units (CFU) throughout the course of the experiment. Every 50 generations, CFU counts were taken for 6 populations from each condition. In all, 10 μL of culture was diluted 10^5^-fold into 1x PBS and plated onto selective YPD agar–cycloheximide to select for *L. plantarum* and G418 to select for *S. cerevisiae* for each dilution. Plates with a suitable number of colonies were recorded and used to calculate CFU/mL.

### Fitness assays

Fitness assays for both evolved *S. cerevisiae* and *L. plantarum* were conducted for every population in monoculture (*n* = 48) and co-culture (*n* = 96) by comparing population density after 48 hours of growth. Strains coming from the −80 ^o^C freezer were passaged by diluting 32-fold in a 96-well plate to re-acclimatise over 48 hours of growth. Populations were passaged into fresh media again, but separated by species using selective media for *L. plantarum* (cycloheximide) and *S. cerevisiae* (G418) in separate 96-well plates and grown under experimental conditions for 48 hours. Growth plates were then shaken at 1000 rpm and then measurements of optical density (600 nm) were taken.

### Whole-genome sequencing

DNA was extracted using GenFind v3 kit (Beckman Coulter). Modifications for the enzymatic lysis were made as follows: *S. cerevisiae* lyticase (Sigma, 1000 U/ml) for 60 min. at 30 ^o^C, cells were pelleted and resuspended in proteinase K and RNAse A (100 mg/mL) and incubated at 50 ^o^C for 60 min. For *L. plantarum* lysozyme (Sigma, 100 mg/ml) for 60 min. at 37 ^o^C followed by proteinase K and RNAse A as above. DNA concentrations were measured on a Quantus analyser (Promega) before library preparation using Illumina DNA Prep kit (Illumina) using a modification manufacturer’s instructions to reduce reaction volumes to 25% of the recommended amount. Libraries were sequenced on the NextSeq 550 (Illumina) platform, using a Mid Output Kit v2.5 (150 cycles) [39]. An appropriate reference for the *S. cerevisiae* strain YJM978 was available (Project accession number: PRJNA189874; [40]): however, *de novo* assembly of the *L. plantarum*, short reads revealed no available reference sequences appropriate for use in reference-guided assembly. Therefore, to generate a reference sequence for the *L. plantarum* ancestor, DNA was prepared for long-read sequencing with the MinION device (Oxford Nanopore Technologies) according to protocols previously outlined [41]. Long reads were de-multiplexed and base-called according to Wick et al. [42]. The Unicycler v0.4.4 and Bandage v0.8.1 software packages, respectively, were used for hybrid assembly of short and long reads [39], and visualisation thereof [43]. The presence of a series of long repetitive regions in the bacterial chromosome precluded its complete assembly, but resulted in the contiguous assembly of 96% (3,029,606 bp) of the chromosome, with the remainder predicted to represent a triplicated region of 40,495 bp (triplicate based on mean read depth relative to that of the long contig). The sequences of two potential plasmids (9211 and 3493 bp in length) carried by the ancestral *L. plantarum* stain were also resolved as circular replicons, although it cannot be ruled out that these forms extended repeat regions within the LPKH chromosomal genome (Accession number PRJNA749634). Annotation of the hybrid assembly was completed using RAST [44]. Open-reading frames from RAST annotation underwent BLAST searches to look for homologous gene names; if a gene homologue was found with 100% sequence similarity in either *Escherichia coli* or *Bacillus subtilis*, then the gene name was amended to the RAST annotation. The completed *L. plantarum* sequence was denoted strain LPKH. Both the nearly assembled sequence and the short and long reads from sequencing were uploaded to NCBI databases under the BioProject number PRJNA749634.

After 925 generations, 10 evolved populations from each treatment were chosen for whole-genome sequencing. About 10 μL of each population was grown to saturation in modified CSM, with co-cultured samples grown in separate antibiotic selective media with either cycloheximide or G418 to isolate *L. plantarum* and *S. cerevisiae*, respectively. DNA preparation and short-read sequencing for the evolved populations was performed as described above. Resequencing of the evolved populations using either the ancestral *L. plantarum* or *S. cerevisiae* genome as appropriate was performed with *breseq* v.0.33.2 in polymorphic mode. [45]. Each sample was sequenced to a depth of ~200× coverage for *L. plantarum* and ~50× coverage for *S. cerevisiae*. The gdtools suite of programs for processing *breseq* output files was used to determine which mutations were unique or shared across monoculture and co-culture populations [45]. Intergenic mutations were not considered in downstream analyses for either species. Single-nucleotide polymorphisms at or above 5% frequency in *L. plantarum* and 30% frequency for *S. cerevisiae* were used for analysis of genomic alterations, corresponding to 10 reads for each variant. Populations were labelled with the well isolated from and prefixed with the treatment of either C for co-culture or M for monoculture (e.g., population M2D was isolated from well 2D on the monoculture 96-well plate).

### Multi-hit genes and parallel evolution analysis

The independent parallel evolution of a trait is often taken as evidence of an adaptive effect of that trait [46, 47]. Parallel evolution can occur at the level of the gene in microbial evolution experiments and can suggest the action of selection [48**–**51]. Here, genes that have evolved mutations in independent replicate populations are known as “multi-hit” genes. To test the hypothesis that our multihit genes were the targets of natural selection and not the result of neutral evolutionary processes, we generated a null model using an in-house python script to simulate the number of multihit genes in our experiment, given no natural selection. For each species, the NumPy [52] function ‘random.choice’ was used to create a set of elements, the number of which is equal to the number of protein-coding genes in *L. plantarum* (3006) and *S. cerevisiae* (5446). The probability of each element being selected was weighted by the gene lengths. A number of draws were made from each set of elements, matching the number of called (observed) mutations. For the parallel-evolution analysis, we used the same criteria for “called mutations” described above, except that we excluded identical mutations that occurred in more than one population (except for the first instance) and in the case where a single population had more than one mutation in the same gene, we counted this as only a single “hit”. The process of drawing mutations was repeated 10,000 times for each analysis, allowing us to generate statistical significance for each multihit gene. We judged our experimental results to be significantly different from the null model if the number of times a gene is hit in our experiment was greater than the maximum number of hits in any one of 10,000 simulations. We defined multihit genes as those that acquired mutations in the open-reading frames of genes in two or more replicate populations during the course of the evolution experiment, providing that number is more than predicted from the null model. Using this model, we also compared if natural selection was acting more generally in our environments, and not just on a gene-by-gene level. From our whole-genome sequencing, we took all genes that contained at least one non-synonymous mutation, and grouped them based on the number of non-synonymous mutations across all 10 populations per treatment in both species. Average gene size was calculated for each group. For the null model, a similar process was applied, except groups were determined by the maximum number of hits in each gene across 10,000 repeats of the null model.

### Invasion assays

The ecological stability of our ancestral ecosystem was tested with reciprocal invasion assays. Single colonies of both species were grown to saturation in 132 μL of CSM in a foil-sealed 96-well plate (*n* = 12) for 48 hours. In all, 10x and 100x diluted stocks were then created for *L. plantarum* and *S. cerevisiae* using 1x phosphate-buffered saline. Diluted stocks of one species were then mixed with undiluted cultures of the other species in 1:1, 9:1 and 1:9 ratios (undiluted culture:diluted culture) to create mixes with a wide range of starting ratios, ranging from 1:200 to 200:1 (*L. plantarum*: *S. cerevisiae*). About 4 μL of each mix was placed into 128 μL of CSM (*n* = 3 per mix) and grown under experimental conditions for 10 days (5 transfer cycles). At the end of each transfer cycle, CFU was counted by plating cultures onto selective agar. CFU counts were combined to create a ratio of *L. plantarum*: *S. cerevisiae*.

For assessment of ecological stability in our monoculture- and co-culture-evolved strains, the same protocol was used, with the following changes. Ancestral, three monoculture-evolved and three co-culture-evolved *L. plantarum* strains were mixed with an ancestral, monoculture evolved and co-culture evolved *S. cerevisiae* in populations where each species was both common and rare (*n* = 3 each). CFU counts were taken for all cultures before mixing, to measure initial inoculum densities, and after 48 hours of growth under experimental conditions to measure the final culture densities. To quantify fitness and niche differences, a protocol outlined in Zhao et al. 2015 was adopted [53]. Fitness differences between each pairing of *L. plantarum* (LP) and *S. cerevisiae* (Y) were measured as the difference in population densities (K) when grown in monoculture, as described by the following equation [53]:

$${{{{{\rm{fitness}}}}}}\,{{{{{\rm{difference}}}}}} = \log _{10}\left( {\frac{{K_{LP}}}{{K_Y}}} \right)$$

Larger values indicate greater fitness differences. Niche overlap was assessed based on reciprocal invasion tests. Each pairing of *L. plantarum* and *S. cerevisiae* were grown in two treatments, one in which *L. plantarum* is common and *S. cerevisiae* is rare (Treatment C), and one in which *S. cerevisiae* is common and *L. plantarum* is rare (Treatment R). Initial frequencies (*P*) of each species were recorded (e.g., *P*_LP-C_ for the initial frequency of *L. plantarum* in Treatment C, and *P*_LP-R_ for the initial frequency of *L. plantarum* in Treatment R). A Malthusian parameter was calculated for each species in each treatment, as described by the following equation:

$$m = \ln \left( {\frac{{Nf}}{{Ni}}} \right)$$

where *N*i and *N*f are the initial and final densities, respectively [54]. Relative growth rates of each species in each treatment were measured as a selection coefficient (*S*), as described by the following equation [53]:

$$S_{{{{{\rm{LP}}}}}} = m_{{{{{\rm{LP}}}}}} - m_Y$$

$$S_Y = m_Y - m_{{{{{\rm{LP}}}}}}$$

Niche overlap was then assessed by examining the effect of initial inoculum density on the relative growth rate of each species, using the following equation:

$${{{{{\rm{niche}}}}}}\,{{{{{\rm{overlap}}}}}} = \frac{{S_{{{{{{\rm{LP}}}}}} - C} - S_{{{{{{\rm{LP}}}}}} - R}}}{{P_{{{{{{\rm{LP}}}}}} - C} - P_{{{{{{\rm{LP}}}}}} - R}}}$$

Here, the relative differences in selection coefficient are normalised based on their initial frequency in the population [53]. The calculated values for all evolved pairings were then normalised based on the niche overlap of the two ancestral strains; a more negative value than the ancestral pairing suggests a decrease in niche overlap over the course of our evolution experiment, and a more positive value indicates an increase in niche overlap. In short, niche difference is calculated as the reduction in carrying capacity of each strain when grown from the least common species compared with when grown from the most common species; if strains have overlapping niches, then carrying capacity will be reduced for either species when grown from rare. However, if strains are in separate niches, then there will be no difference in the carrying capacity of each strain when grown from common or grown from rare. The strength of coexistence was based on the smallest selection coefficient from rare for each pairing when initially rare (*S*_LP-R_, *S*_Y-R_), as both species must be able to invade from rare to qualify as coexistence.

### Competitive growth assays

The ecological stability of our evolved strains in pairwise ecosystems was tested using competitive growth assays. Single colonies of ancestral, monoculture (*n* = 3) and co-culture (*n* = 3) strains of both species were grown to saturation separately in 132 μL of CSM in a foil-sealed 96-well plate for 48 hours. About 10 μL of each strain was mixed with 10 μL of every other strain of the other species in separate wells. About 4 μL of each mix was placed into 128 μL of CSM (*n* = 4 per mix) and grown under experimental conditions for 20 days (50 generations, 10 transfer cycles). After 3, 4, 5 and 10 days (15, 20, 25 and 50 generations, respectively), CFU was counted by plating cultures onto selective agar to track the abundance of each species.

For pairwise co-cultures with reduced glucose, the same protocol was conducted, with the following changes. Co-culture-evolved *S. cerevisiae* was used to pair with ancestral, monoculture and co-culture *L. plantarum*. About 4 μL of each mix was placed into 128 μL of CSM with 2% glucose (experimental standard), 1%, 0.5% and 0.25% glucose (*n* = 3 each). In total, 96-well plates were incubated under experimental conditions for 3 transfer cycles (15 generations), with CFU counts taken for each transfer cycle. The difference between the initial and final CFU of each *L. plantarum* strain was used to calculate a selection coefficient in either high (1% and above glucose) or low (below 1%) sugar concentrations when paired with ancestral or coevolved *S. cerevisiae*.

### Statistical methods

All statistical tests conducted were one-way ANOVA with Bonferroni-corrected post hoc *t*-tests performed in GraphPad Prism version 9.0.0 for MacOS (GraphPad Software, San Diego, California USA, www.graphpad.com), unless otherwise stated.

## Results

### *L. plantarum* and *S. cerevisiae* are ecologically stable in co-culture and occupy distinct niches

**Fig. 1:**
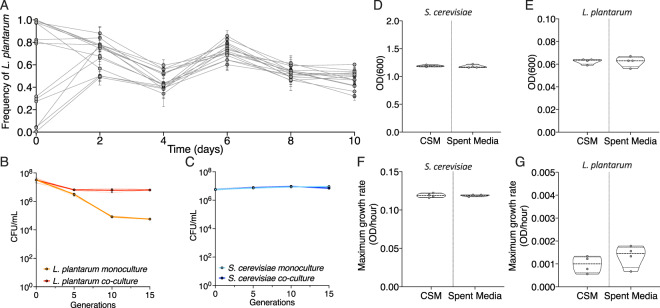
*L. plantarum* and *S. cerevisiae* are stable in co-culture. **A** The frequency of *L. plantarum* in co-culture with *S. cerevisiae* co-culture over 10 days, initiated at 15 different starting ratios of *L. plantarum:S. cerevisiae*. Measurements were taken at 2-day intervals. Each point shows the average of three independent replicates. The population carrying capacity of ancestral (**B**) *L. plantarum* and (**C**) *S. cerevisiae* over 15 generations in monoculture and co-culture. Carrying capacity (optical density) and growth rates of the ancestor *S. cerevisiae* and *L. plantarum* strains after 48 hr of growth in Complete Supplement Media (CSM) and CSM media that had already been spent by the other species (**D–G**).

We obtained an isolate of *L. plantarum* from a sourdough bread culture (Methods), established laboratory cultures and carried out reciprocal invasion assays to determine whether *L. plantarum* and *S. cerevisiae* could establish an ecologically stable co-culture. We founded cultures with a range of frequencies, ranging between 99:1 and 1:99 (*L. plantarum*: *S. cerevisiae*). After passaging for 10 days (5 transfers), we found that all replicate co-cultures (*n* = 15) converged upon a stable equilibrium frequency, with an average *L. plantarum* frequency of 47.7% (95% Confidence Interval (CI) [44.8%, 50.7%], range [22.2–64.7%]) (Fig. 1A). Neither species went extinct in any of our pairings.

Next, we compared the performance of the two species in co-culture and monoculture. We grew our ancestral *L. plantarum* and *S. cerevisiae* strains in either co-culture or monoculture for 15 generations, a short enough period of time that the population is unlikely to evolve, but that should reveal cumulative effects of ecological interactions over multiple transfers in batch culture. We found a significant reduction of population size in our monoculture *L. plantarum* populations when grown in monoculture compared with co-culture (unpaired t-test, *p* < 1 × 10^−^^4^) (Fig. 1B), which suggests that *L. plantarum* in monoculture may experience different selective pressures than co-culture evolved *L. plantarum*. Conversely, *S. cerevisiae* has a marginal, but significant reduction in population size when in co-culture compared with monoculture (unpaired *t*-test, *p* = 0.021) (Fig. 1C). Overall, this indicates that *L. plantarum* growth is supported by co-culture with *S. cerevisiae*.

Given this potential interaction, we quantified the fitness and niche difference between our two species. Co-existence theory predicts that ecologically stable species will have largely non-overlapping niches, or small differences in fitness [34]. We grew our ancestral *L. plantarum* and *S. cerevisiae* strains in both experimental growth media and growth media that had been “spent” by growing the other species to carrying capacity and then removing the cells (Methods). We hypothesised that if *L. plantarum* populations attained the same culture density in the spent media of *S. cerevisiae* as when they were grown in isolation, *L. plantarum* must utilise different resources from the growth media than *S. cerevisiae*, or that *L. plantarum* benefits from products excreted by *S. cerevisiae*. However, if there is a reduction in growth performance in spent media compared with unspent media, this suggests that there is niche overlap between the two species. We found no significant difference in either maximum optical density (unpaired *t*-test, *t* = 0.55, *p* = 0.60), or maximum growth rate for *S. cerevisiae* (unpaired *t*-test, *t* = 0.15, *p* = 0.88) (Fig. 1D & F), or *L. plantarum* (unpaired *t*-test (maximum optical density) *t* = 0.097, *p* = 0.93; unpaired *t*-test (maximum growth rate) *t* = 1.2, *p* = 0.28) (Fig. 1E & G) when grown in spent media rather than experimental media (CSM) (Supplementary Fig. 1). This indicates that both species occupy distinct niches in experimental media. Initial fitness differences in our experimental media were also quantified by comparing the maximum optical density each species attains in experimental media (alone) over 48 hours. Overall, our initial phenotyping of *L. plantarum* and *S. cerevisiae* pairwise communities shows that our species have large fitness and niche differences, but are ecologically stable in co-culture. This supports a commensal (+, 0) relationship between the two species.

### *S. cerevisiae* constrains *L. plantarum* evolution in co-culture

**Fig. 2:**
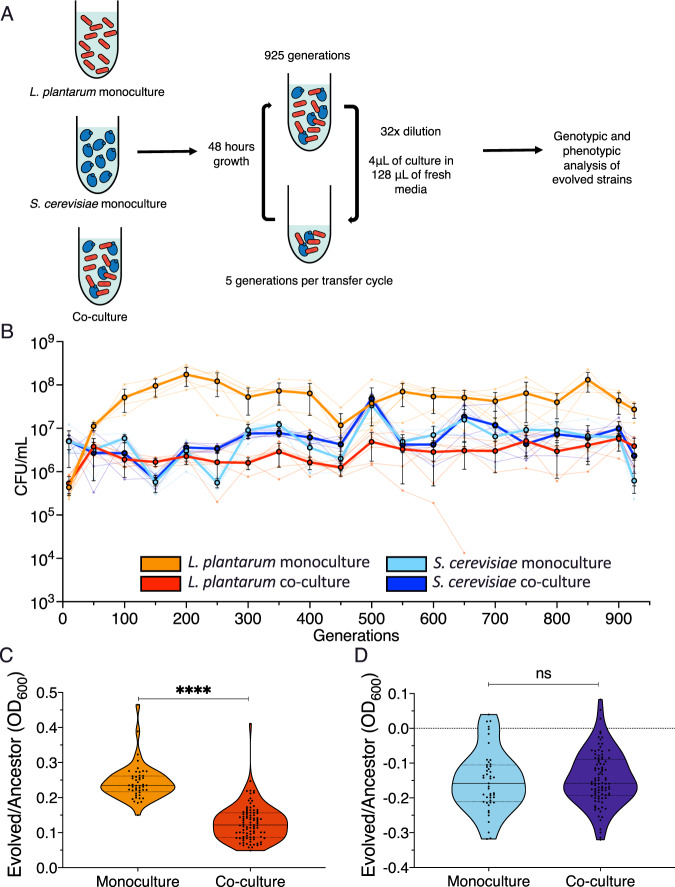
*L. plantarum* adaptation is limited by co-culture with *S. cerevisiae*. **A** Replicate populations of *L. plantarum* (red) and *S. cerevisiae* (blue) were propagated in either monoculture or co-culture conditions for 925 generations. **B** Population carrying capacity estimated by colony-forming units (CFU) per mL of *L. plantarum* and *S. cerevisiae* in monoculture and co-culture over 925 generations. Measurements were taken at 50 generation intervals. Each point shows the average of six independent evolution experiments. Growth assays of (**C**) *L. plantarum* and (**D**) *S. cerevisiae* after 925 generations of evolution, measured as the relative difference in optical density compared with the ancestral strain. Error bars show the S.E.M.

To study the outcomes of each species evolving in the absence of the other, we propagated replicate populations of *L. plantarum* and *S. cerevisiae* in monoculture and co-culture treatment conditions. The monoculture treatment consisted of growing each species in the same growth conditions but separately from the other. Co-culture treatment involved propagating both species together in the same container (Fig. 2A). We tracked the population sizes of both species in each condition by counting colony-forming units (CFU) of a few sample populations across 925 generations (Fig. 2B). After 925 generations, 95 out of 96 co-culture populations contained both *L. plantarum* and *S. cerevisiae*, with a single case of *L. plantarum* going extinct after ~700 generations. These results suggest that our co-culture environment promotes the stable coexistence of both species.

In our monoculture-evolved *L. plantarum* populations, we observed a rapid increase in carrying capacity after only 100 generations of evolution, rising from ~4 × 10^5^ CFU/mL after 10 generations to ~2 × 10^8^ CFU/mL after 200 generations (Fig. 2B). This remains significantly higher than the carrying capacity seen in co-culture with *L. plantarum* across the entire course of the experiment (unpaired *T*-test, *t* = 12.56, *p* < 1 × 10^−^^4^) (Fig. 2C). In contrast, we observed no significant difference in the carrying capacity between monoculture- or co-culture-evolved *S. cerevisiae* (unpaired *T*-test, *t* = 0.52, *p* = 0.60) (Fig. 2D). Altogether, these data suggest that *L. plantarum* evolution is constrained by *S. cerevisiae* in co-culture, and that the absence of *S. cerevisiae* results in the ecological release of *L. plantarum* populations.

### Ecological release alters the genetic targets of selection in evolving populations of *L. plantarum*

To determine the genetic causes of adaptation, we sequenced 10 of each of the *L. plantarum* and *S. cerevisiae* populations from monoculture and co-culture treatments after 925 generations of evolution. We first looked for an effect of mutation class and experimental treatment on the total number of mutations that evolved, and found no significant difference across *L. plantarum* populations (two-way ANOVA, *L. plantarum*, *F*_(mutation class)_ = 68.72, *p* < 1 × 10^−^^4^; *F*_(experimental treatment)_ = 0.19, *p* = 0.67) (Supplementary Fig. 2). *S. cerevisiae* monoculture populations showed a significant increase in the total number of mutations and the number of coding-region mutations compared with co-culture populations (two-way ANOVA, *S. cerevisiae*, *F*_(mutation class)_ = 65.59, *p* < 1 × 10^−^^4^, *F*_(experimental treatment)_ = 14.56, *p* < 0.001).

**Fig. 3:**
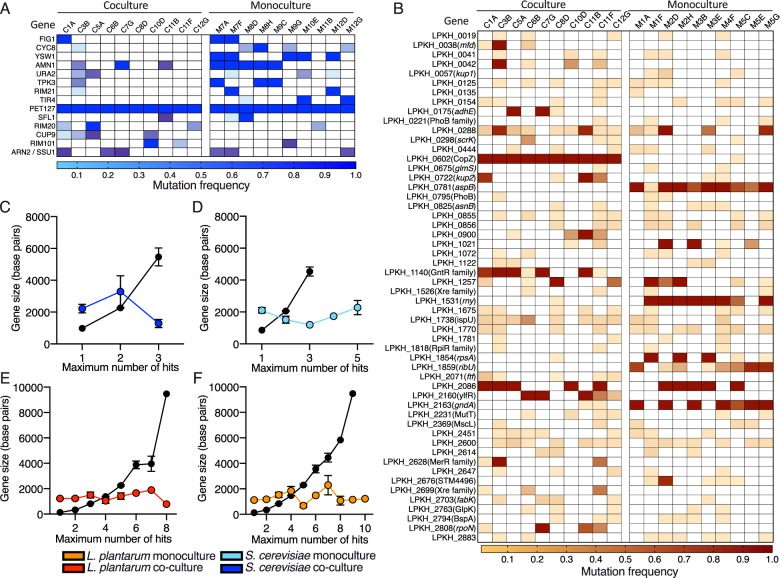
The genetic targets of natural selection in monoculture and co-culture-evolved *L. plantarum* and *S. cerevisiae*. The frequency of non-synonymous mutations (rows) in 10 co-culture-evolved and 10 monoculture-evolved populations (columns) of (**A**) *S. cerevisiae* and (**B**) *L. plantarum*. Populations were labelled with the well isolated from and prefixed with the treatment of either C for co-culture or M for monoculture. Genes with mutations in at least three replicate populations, with at least one of those mutations at a frequency > 0.2, are shown. In co-culture *L. plantarum*, one highly parallel genetic target, LPKH_0602(CopZ), contains the exact same mutation in all 10 co-culture populations, indicating that these mutations originated as standing genetic variation, rather than *de novo* mutations that evolved during the experiment. Keys below each graph indicate the frequency of each mutation; darker colouration corresponds to mutation at greater frequency. (**C**–**F**) The number of genes “hit” across replicate populations is compared with expectations under a null model of no natural selection (black line) that takes gene length into account since longer genes are more likely than shorter genes to be hit by mutation. The coloured lines and markers show the observed number of genes hit X times for (**C**) co-culture *S. cerevisiae*, (**D**) monoculture *S. cerevisiae*, (**E**) co-culture *L. plantarum* and (**F**) monoculture *L. plantarum*. Error bars displayed represent S.E.M.

Parallel evolution is evidence for natural selection [55], so we looked for genes that had evolved mutations across multiple replicate populations during the evolution experiment. We identified “multi-hit” genes that sustained mutations more often than expected under a null model of evolution that takes gene length into account (Methods). Overall, we found 28 multihit genes in *S. cerevisiae* (Fig. 3A), and 8 multihit genes in *L. plantarum* (Fig. 3B) populations (Supplementary Table S1 and S2, respectively). We observed stronger signatures of parallel evolution in monoculture treatment than co-culture treatment populations for both *S. cerevisiae* and *L. plantarum* (Fig. 3D–F). The targets of selection were similar across *S. cerevisiae* populations (Fig. 3A). In *L. plantarum*, we found 12 multi-hit genes identified in co-culture populations that were not hit in a single monoculture population, and six multihit genes in monoculture populations that were not hit in co-culture populations (Fig. 3B). This suggests that there are distinct genetic targets of evolution of monoculture- and co-culture-evolved *L. plantarum*.

### The evolutionary loss of ecological stability in monoculture-evolved *L. plantarum*

**Fig. 4:**
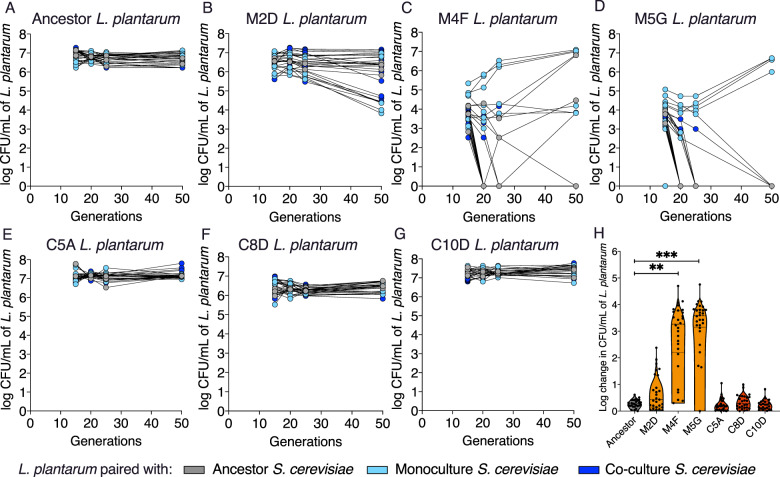
The evolutionary loss coexistence in monoculture-evolved *L. plantarum*. Colony forming units (CFU) per mL of (**A**) ancestral, (**B**–**D**) monoculture and (**E–G**) co-culture *L. plantarum* at 15, 20, 25 and 50 generations. All strains were co-cultured with the ancestor *S. cerevisiae* strain, three monoculture-evolved and three co-culture-evolved *S. cerevisiae* strains. Points represent all replicates of all pairings. *L. plantarum* strains are labelled with their experimental treatment and position in the microplate. For example, M2D is a monoculture-evolved *L. planatarum* population from microplate well 2D, and C5A is a co-culture-evolved *L. plantarum* population from microplate well 5A (one-way ANOVA, *p* < 0.0073 and *p* < 2 × 10^−4^, respectively, *F* = 6.78), with no significant change seen in co-cultured *L. plantarum* strains (one-way ANOVA, *p* > 0.99, *F* = 6.78). The carrying capacity (CFU) at generation 10 subtracted from generation 50 (**H**). Two monoculture *L. plantarum* populations - M4F and M5G - show a significant log change compared to the ancestral *L. plantarum* strain (one way ANOVA, *p* < 0.0073 and *p* < 0.0002 respectively, *F* = 6.775), with no significant change seen in co-cultured *L. plantarum* strains (one way ANOVA, *p* > 0.999, *F* = 6.775).

To test if our monoculture-evolved strains retained the ability to coexist with the other species, we initiated co-cultures with pairwise combinations of ancestor-, monoculture-, and co-culture-evolved strains (Fig. 4), and tracked their population densities over 50 generations. We found that ancestor- and co-culture-evolved *L. plantarum* populations were able to maintain stable frequencies in co-culture with *S. cerevisiae*, regardless of the *S. cerevisiae* strain background with which it was paired. In contrast, monoculture-evolved *L. plantarum* went extinct or decreased in frequency when paired with *S. cerevisiae*. For example, *L. plantarum* strains M4F and M5G are highly unstable in co-culture, with only eight and four of the initial 28 combinations remaining in co-culture after 50 generations, respectively (Fig. 4C, D). We compared the change in CFU/mL between the first and last timepoints, and found that two of the three monoculture *L. plantarum* strains—M4F (one-way ANOVA, *F* = 6.77 Bonferroni test, *t* = 3.26, *p* < 0.0080) and M5G (one-way ANOVA, *F* = 6.77 Bonferroni test, *t* = 4.25, *p* < 2 × 10^−4^)—show a significant change compared with the ancestral *L. plantarum* strain (one-way ANOVA, *F* = 6.77, Bonferroni test, *t* = 0.013, *p* > 0.99). In contrast, no significant change was seen in the three co-cultured *L. plantarum* strains (one-way ANOVA, *F* = 6.77, Bonferroni test, C5A *t* = 3.50 × 10^−^^5^, C8D *t* = 4.63 × 10^−4^, and C10D *t* = 5.88 × 10^−^^5^, *p* > 0.99 for all three) (Fig. 4H). Although none of the monoculture-evolved *L. plantarum* lineage M2D co-cultures were driven extinct by *S. cerevisiae*, we did observe a reduction in average population size after 50 generations, from 4.45 × 10^6^ (CI [3.27 × 10^6^, 5.63 × 10^6^], minimum 4 × 10^5^, maximum 1.20 × 10^7^) to 3.56 × 10^6^ (CI [2.03 × 10^6^, 5.09 × 10^6^], minimum 6.67 × 10^3^, maximum 1.47 × 10^7^). Altogether, these results suggest that the ecological release of *L. plantarum*, and its subsequent adaptation to monoculture growth conditions, drives the evolutionary loss of ecological stability.

### Reduced invasive ability of monoculture *L. plantarum* in lower glucose concentrations

The most frequently selected non-synonymous or nonsense mutations in monoculture-evolved *L. plantarum* populations occurred in three genes that have key roles in carbon metabolism: LPKH_153 (*rny*) (ribonuclease Y), LPKH_2163(*gndA*) (phosphoglutarate dehydrogenase activity) and LPKH_0781(*aspB*) (aspartate aminotransferase activity) [56, 57] (Supplementary Fig. 3). These genes are not well-studied in *L. plantarum*, but are highly conserved. LPKH_0781(*aspB*) codes for aspartate aminotransferase, an enzyme important in carbon metabolism, which links fermentation and nitrogen metabolism via pyruvate and aspartate [58]. LPKH_1531 (*rny*) codes for ribonuclease Y, an mRNA degradation enzyme involved in post-transcriptional regulation of gene expression [59]. LPKH_2163 (*gndA*) codes for 6-phosphogluconate dehydrogenase, a key enzyme in the oxidative phase of the pentose phosphate pathway [60]. The mutations in *rny* were all non-synonymous substitutions, suggesting that these mutations modified *rny* function. However, *gnd* and *aspB* evolved nonsense or early stop mutations, suggesting that a partial or total loss of function of these genes is beneficial for *L. plantarum* in monoculture growth conditions.

**Fig. 5:**
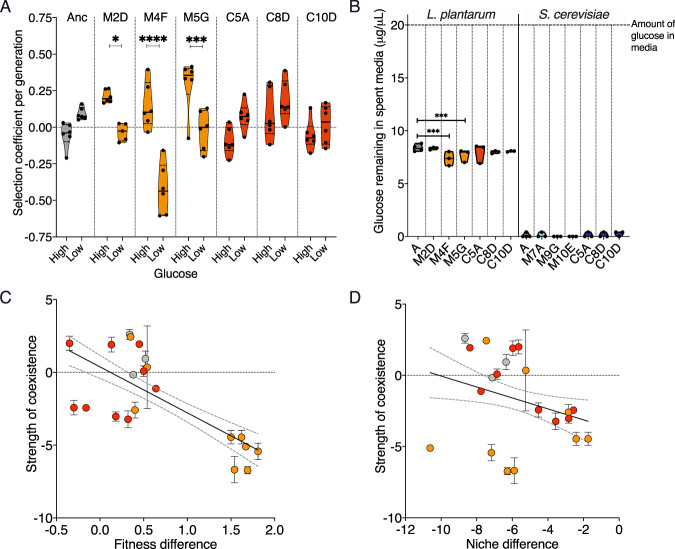
Evolved fitness differences drive the breakdown in coexistence between *L. plantarum* and *S. cerevisiae*. **A** Fitness of ancestor- (grey), monoculture- (orange) and co-culture- evolved (red) *L. plantarum* when paired with coevolved *S. cerevisiae* in growth media with high or low amounts of glucose. *L. plantarum* strains are labelled with their experimental treatment and position in the microplate. For example, M2D is a monoculture-evolved *L. plantarum* population from microplate well 2D, and C5A is a co-culture-evolved *L. plantarum* population from microplate well 5A. **B** Amount of glucose remaining in spent media of ancestral-, monoculture- and co-culture-evolved *L. plantarum* and *S. cerevisiae* cultures after 48 hours. The relationship between the strength of coexistence with (**C**) fitness difference (*R*^*2*^ = 0.48, *p* < 1 × 10^−^^4^) and (**D**) niche difference for *L. plantarum* and *S. cerevisiae* pairwise communities (*R*^2^ = 0.085, *p* = 0.021). All error bars represent standard deviation. Orange dots represent monoculture-evolved *L. plantarum* strains, red dots represent co-culture-evolved *L. plantarum* strains.

Since glucose, the preferred carbon source of *S. cerevisiae*, is the most abundant carbon source in CSM growth media, we tested whether monoculture-evolved *L. plantarum* had evolved an increased dependence on glucose compared with the *L. plantarum* ancestor. To do this, we propagated *L. plantarum* and *S. cerevisiae* in experimental media with a range of glucose concentrations (Methods). Here, we paired our ancestral-, monoculture-, and co-culture-evolved *L. plantarum* strains with either ancestral or co-cultured *S. cerevisiae*, recorded the population size (CFU) at 10 generations, and calculated a selection coefficient for each strain based on its capacity to invade that specific *S. cerevisiae* strain (Fig. 5A). Against the co-evolved *S. cerevisiae* background, all three monoculture-evolved *L. plantarum* strains had significantly reduced selection coefficients in low-glucose concentrations compared with high glucose concentrations (two-way ANOVA, *F*_(glucose concentration)_ = 11.15, *p* > 0.005; *F*_(strain)_ = 7.35, *p* < 1 × 10^−^^4^). Ancestral (*t* = 1.98, *p* = 0.36) and co-culture-evolved (C5A *t* = 2.39, *p* = 0.14; C8D *t* = 1.35, *p* > 0.999; C10D *t* = 1.02, *p* > 0.99) *L. plantarum* had no significant difference in selection coefficient in either high- or low glucose concentrations (Two-way ANOVA, *F*_(glucose concentration)_ = 11.15, *p* > 0.005; *F*_(strain)_ = 7.35, *p* < 1 × 10^−^^4^), (Fig. 5A). To further explore the relationship between glucose- and monoculture- evolved *L. plantarum* strains, we performed a colorimetric glucose-uptake assay using our ancestral and evolved *L. plantarum* and *S. cerevisiae* strains (Methods). We grew the ancestral, monoculture, and co-culture strains in CSM growth media over 48 hours and tested the amount of glucose remaining. First, we confirmed that the ancestral *L. plantarum* could utilise the majority of the growth media’s glucose supply in 48 hours. This was surprising since our measurements of glucose in growth media that had been “spent” by *S. cerevisiae* revealed no detectable glucose, yet *L. plantarum* grows just as well in this spent media (Fig. 1, Supplementary Figure 1). We also found that two of the three monoculture *L. plantarum* strains (M4F and M5G) that grew contained significantly less glucose than media that had contained the ancestral *L. plantarum* strain (one-way ANOVA, *F*_(*L. plantarum* strain)_ = 10.87; Bonferroni test, M2D *t* = 1.140, *p* > 0.99; M4F *t* = 5.86, *p* = 0.0002; M5G *t* = 6.38, *p* = 1 × 10^−4^) (Fig. 5B). In contrast, there was no significant difference in the amount of glucose remaining in the spent media of ancestral and co-cultured *L. plantarum* strains (one way ANOVA, *F*_(*L. plantarum* strain)_ = 10.87 Bonferroni test, C5A *t* = 2.9, *p* = 0.069; C8D *t* = 2.84, *p* = 0.079; C10D *t* = 2.399, *p* = 0.19) (Fig. 5B). However, the magnitude of these differences in glucose utilisation was small compared with the total amount of glucose consumed, suggesting that the capacity to utilise glucose per se is not the evolutionary cause of the loss of coexistence.

### Fitness differences drive the breakdown of coexistence

Coexistence theory predicts that the evolution of greater fitness differences, or reduced niche differences, between *S. cerevisiae* and *L. plantarum* will increase competitive interactions and reduce the capacity for coexistence [34]. We conducted reciprocal invasion assays to generate estimates of the strength of coexistence, fitness difference and niche overlap for pairs of *L. plantarum* and *S. cerevisiae* [53]. Following Zhao and co-workers, we quantified the strength of coexistence for pairs of *L. plantarum* and *S. cerevisiae* by reciprocal invasion assays (Methods). This rate of invasion for the weaker species was used as the measure for the strength of coexistence. Fitness difference was quantified by measuring the total population carrying capacity of each species when grown in monoculture, and then taking the ratio of the two [34, 53]. The invasion assays were also used to calculate niche overlap; negative values indicate less niche overlap, and positive values indicate greater niche overlap. We found that the best predictor of coexistence was the fitness difference between two strains (*R*^2^ = 0.48, *p* < 1 × 10^−^^4^, Fig. 5C), but niche difference was also able to predict coexistence to a lesser extent (*R*^2^ = 0.085, *p* = 0.021, Fig. 5D).

## Discussion

There are many examples of species evolving new community interactions [7, 17, 61–63]. In this study, we show that evolution outside the constraints of co-culture can drive the evolutionary loss of the capacity to coexist with another species (Supplementary Fig. 4). Previous work has shown that alterations in nutrient availability [2–4] and rapid evolution [5, 6] can destabilise communities. This work shows that positive interactions in ecologically stable communities may not be resilient to adaptation of one of the species to conditions outside of the community context. We further show that not only the magnitude, but also the direction, of evolutionary change determines whether ecological stability will be disrupted.

### Ecological stability lost in monoculture-evolved *L. plantarum* populations

For a pairwise community to be considered ecologically stable, both species must be able to invade the other from rare, i.e., from a lower relative frequency [34]. Previous studies have developed frameworks for predicting how the components of coexistence, such as fitness and niche differences, change during eco-evolutionary processes [64, 65]. Theory predicts that there is a linear relationship between the strength of coexistence and fitness, and a linear relationship between the strength of coexistence and niche differences [66]. If both species can invade the other from rare, one of the species will typically invade the other at a lower rate and take longer to reach equilibrium. For species with non-overlapping niches, the rate of invasion for either species will be the highest when at a low proportion relative to the other species [53]. This is because intraspecific competition is greater than interspecific competition [34], and results in a negative relationship between the rate of increase and the starting relative frequency. For species with overlapping niches, interspecific competition is greater than intraspecific competition, resulting in a positive correlation between growth rate and relative starting frequency [53]. We were surprised to find that increased fitness of *L. plantarum* was the best predictor of the loss of coexistence (Fig. 5). Coexistence theory predicts that an increase in niche overlap will push two species into competition and lead one to exclude the other [33, 34]. Our results suggest that the evolutionary incursion of *L. plantarum* into *S. cerevisiae*’s niche—glucose utilisation—had a minor impact on *S. cerevisiae*. However, it is plausible that a small shift in niche was coupled with a large increase in *L. plantarum* fitness. The loss of coexistence may have been an indirect consequence of *L. plantarum* adaptations to monoculture. For instance, adaptation to glucose media may have resulted in a loss of *L. plantarum’s* capacity to utilise other resources in the growth media. This is supported by the monoculture-selected mutations in *gnd* and *aspB*, which are likely to cause a loss-of-function or truncation. This question will be the subject of future study.

### Low relative fitness drove rapid adaptation in co-culture- and monoculture-evolved *L. plantarum*

During the course of experimental evolution, both species adapted to monoculture and co-culture conditions, although the evolutionary change that we observed was strongly asymmetrical. At the outset of the experiment, *S. cerevisiae* grew well in CSM growth media. After 925 generations, there were very few mutations seen in *S. cerevisiae* populations that were not shared across monoculture and co-culture treatments, and the rate of fitness increase was relatively slow across *S. cerevisiae* populations from both treatments. In contrast, to set up the conditions that supported *L. plantarum* growth, we modified the growth media—CSM was developed for *S. cerevisiae*—by supplementing with amino acids, and incubated the cultures in microaerophilic (non-shaking, sealed) vessels. Despite these efforts, at the beginning of the experiment, *L. plantarum* was poorly adapted to these conditions and attained much lower cell densities than *S. cerevisiae* (Fig. 1). Evolution experiments have consistently found that the lower the fitness of a population, the greater its rate of adaptation [67–72]. It is therefore plausible that the relative maladaptation of *L. plantarum* drove the rapid rate of fitness increase in the monoculture-evolved populations. Although our phenotypic measures of adaptation show that monoculture-evolved *L. plantarum* underwent the most drastic fitness evolution, genome sequencing revealed similarly rapid molecular evolution in the co-culture-evolved *L. plantarum*. A comparison of the high-frequency genetic variants that evolved in *L. plantarum* populations demonstrated that each treatment had its own set of unique multihit genes, confirming that strong selective forces were driving *L. plantarum* adaptation in both monoculture and co-culture conditions. The genetic targets of evolution in the monoculture treatment and competition assays support that *L. plantarum* adapted to high-glucose concentrations by mutations in three key genes involved in carbon metabolism. These data provide evidence that it is not only the strength of selection, but also the trajectory of genetic evolutionary change that can be altered by co-culture with another species, a finding supported by recent monoculture and co-culture evolution experiments [7].

### Co-culture-constrained rapid adaptation preserved ecological stability

The phenotypic and genetic data presented here are evidence that the rapid evolution of *L. plantarum* was not sufficient to drive the evolution of instability, and that the constraining effect of evolution in the presence of *S. cerevisiae* significantly shaped the direction of adaptation. Beneficial mutations are considered to make up a relatively small proportion of all possible mutations [73]. This is because most extant organisms are generally well-adapted to their environment [74, 75], and most mutations that alter the phenotype will reduce fitness [76]. In addition, mutations can have different fitness effects in different environments [77], so it is possible that only a small subset of those mutations that increase fitness for an individual species would also promote stable coexistence with another species. For example, assuming it can escape the drift barrier, a mutation that evolves in a monoculture-evolved *L. plantarum* population that increases the capacity of *L. plantarum* to rapidly utilise glucose, and thereby increases fitness, will be likely to spread and fix in the population. However, in co-culture, if this mutation increases competition between *L. plantarum* and *S. cerevisiae* or is otherwise deleterious, the *L. plantarum* variants containing the mutation would be rapidly outcompeted, given *S. cerevisiae’s* superior capacity to utilise glucose. Co-culture with *S. cerevisiae* therefore constrains the capacity for *L. plantarum* to adapt by limiting it to improvements in fitness based on the use of resources aside from glucose. In contrast, adaptation is not constrained in monoculture-evolved *L. plantarum*. These results are supported by experimental populations of *Pseudomonas fluorescens*, evolved in monoculture or co-culture with a competitor species, *P. putida* [78]. In this study, a mutation that evolved in *P. fluorescens* populations had monoculture-specific fitness effects, supporting that constraining effects of co-culture are not limited to ecologically stable communities.

## Conclusions

There is mounting empirical support that biotic factors—in this case, the presence of *S. cerevisiae*—can drastically shape the phenotypic outcomes of evolution. We found that when *L. plantarum* was evolved separately from *S. cerevisiae*, the capacity for co-culture was lost. Altogether, our data suggest that a pairwise microbial community can preserve ecological stability by constraining the adaptive trajectories of individual species, even if some are maladapted to a recent change in environmental conditions. Conversely, prolonged periods of evolution outside of the community can lead to adaptation that destabilises community structure and ecosystem diversity, even among strains with positive interactions. These results have implications for strategies that employ laboratory evolution to preadapt species or communities to environmental change for reintroduction into the wild [79], and emphasise the importance of incorporating the context of the microbial community into laboratory studies of microbial evolution.

## Supplementary information

Supplementary Figure 1

Supplementary Figure 2.

Supplementary Figure 3

Supplementary Figure 4

Supplementary Figure and Table Legends

## Data Availability

The assembled genome for *Lactobacillus plantarum KH* and raw sequencing reads used to generate the data in Fig. 2 have been deposited in GenBank under the Bioproject ID: PRJNA749634. Custom scripts used for bespoke analyses are available on GitHub (https://github.com/woodlaur189).
