# Supplementary figures and images for: Species interactions constrain adaptation and preserve ecological stability in an experimental microbial community
Source: ISME J. 2022 Jan 22;16(5):1442–52. doi: 10.1038/s41396-022-01191-1 (PMC9039033; doi:10.1038/s41396-022-01191-1)

*S. cerevisiae*

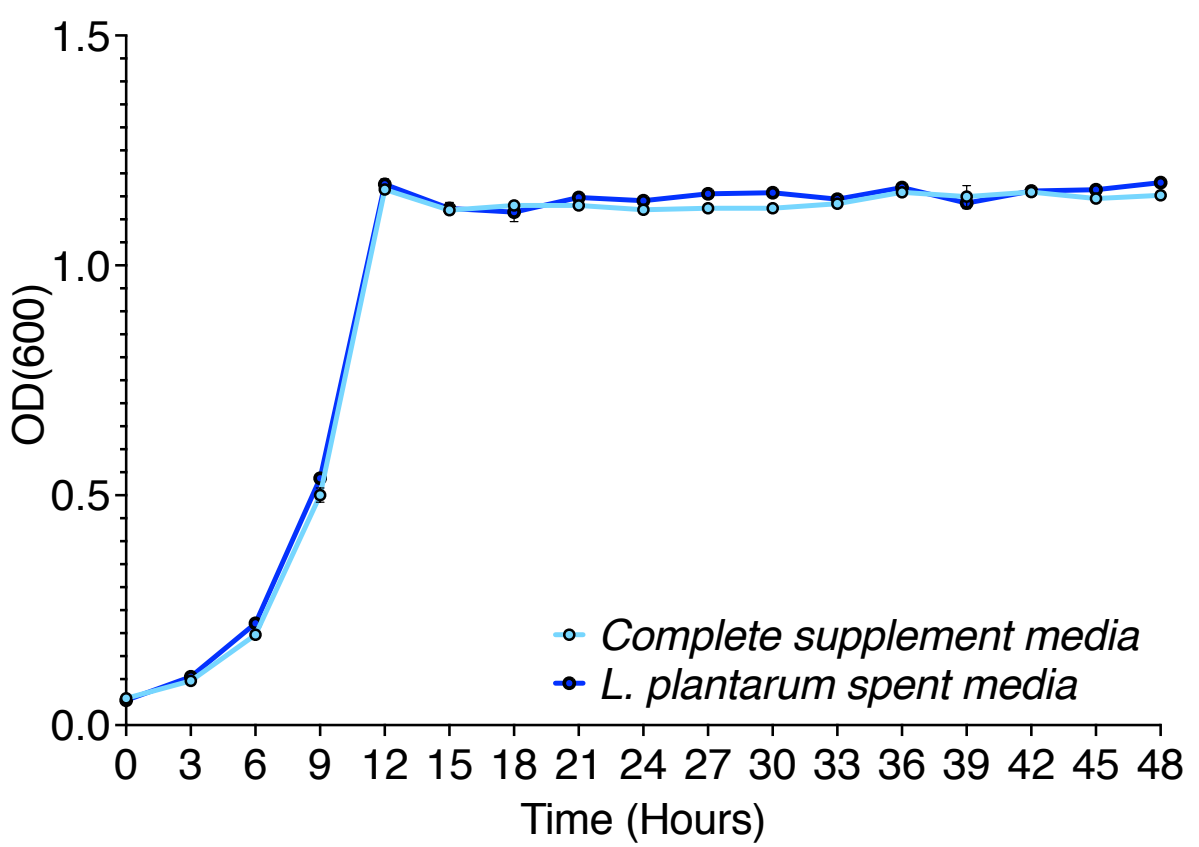

*L. plantarum*

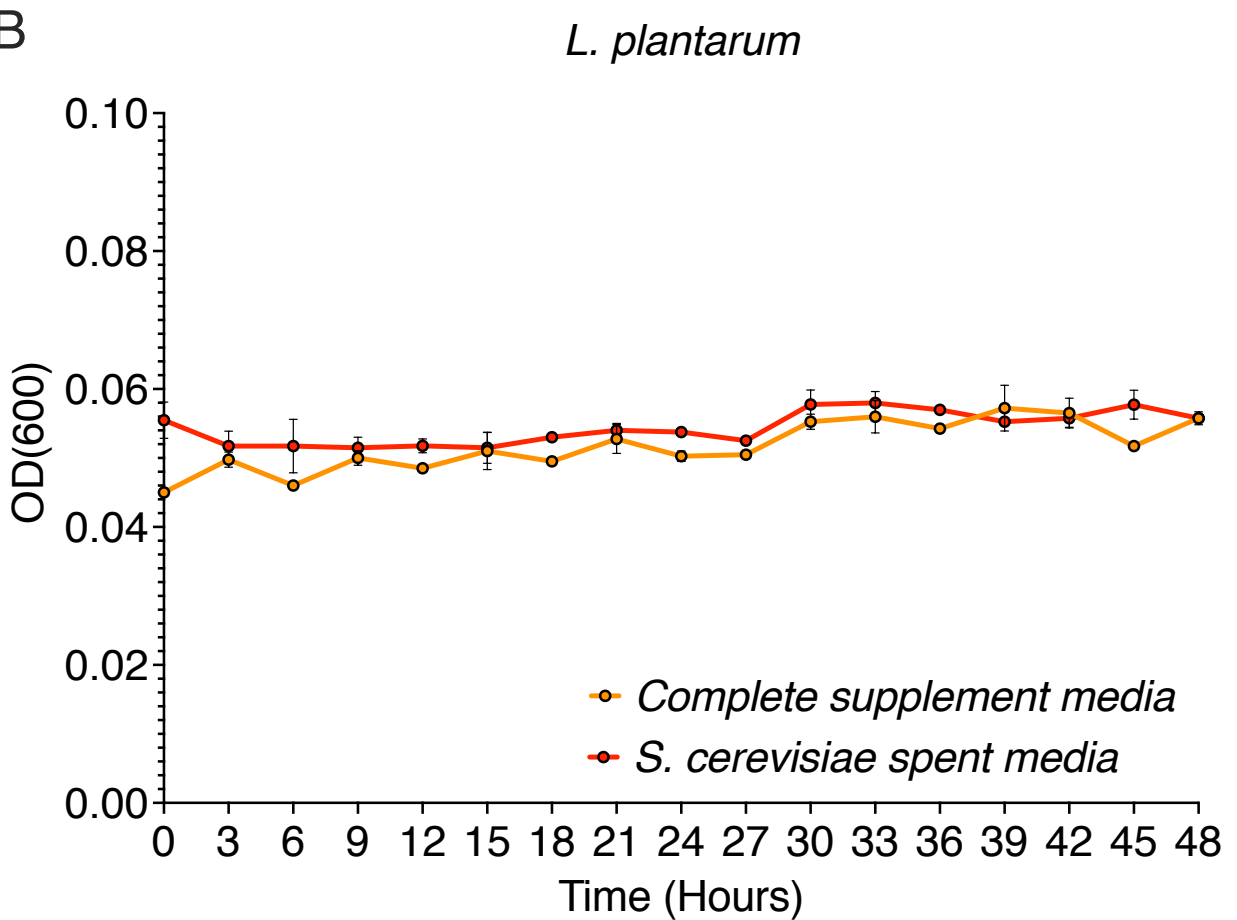

Supplement: Supplementary file 1 — Supplementary Figure 1 [file 41396_2022_1191_MOESM1_ESM.pdf]

*L. plantarum*

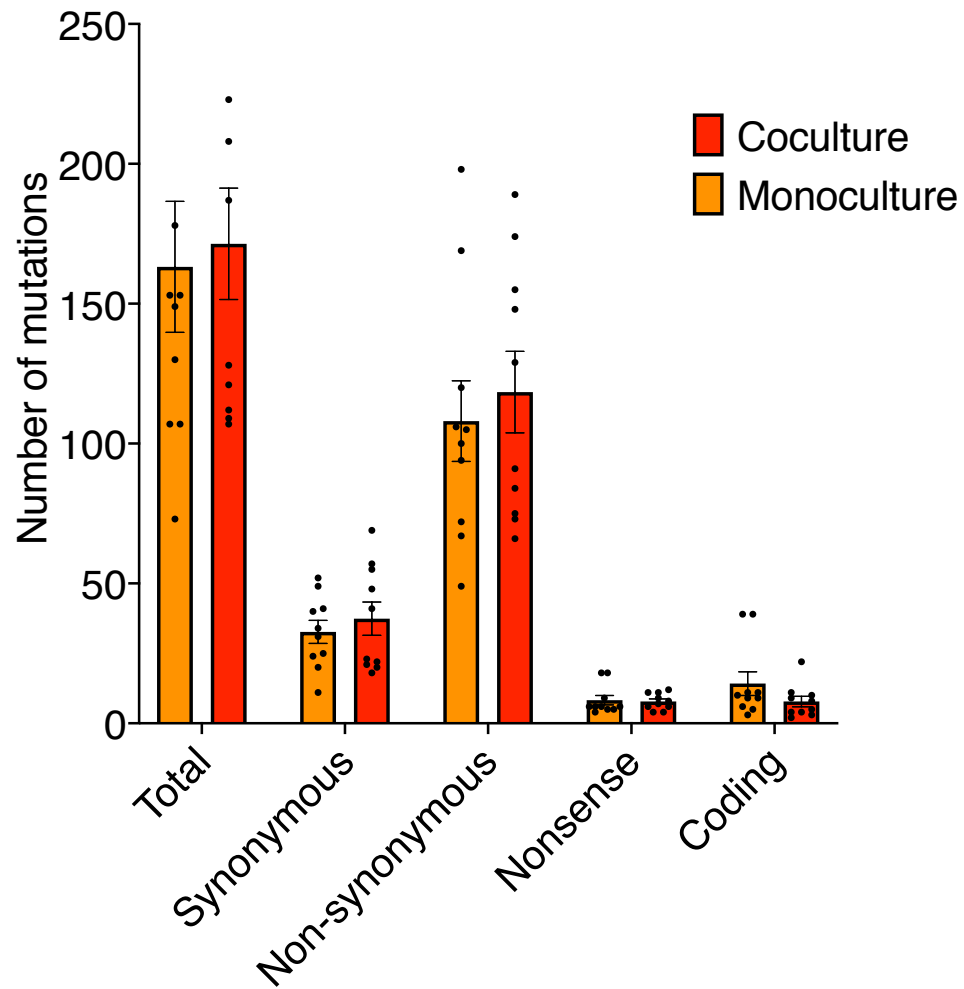

*S. cerevisiae*

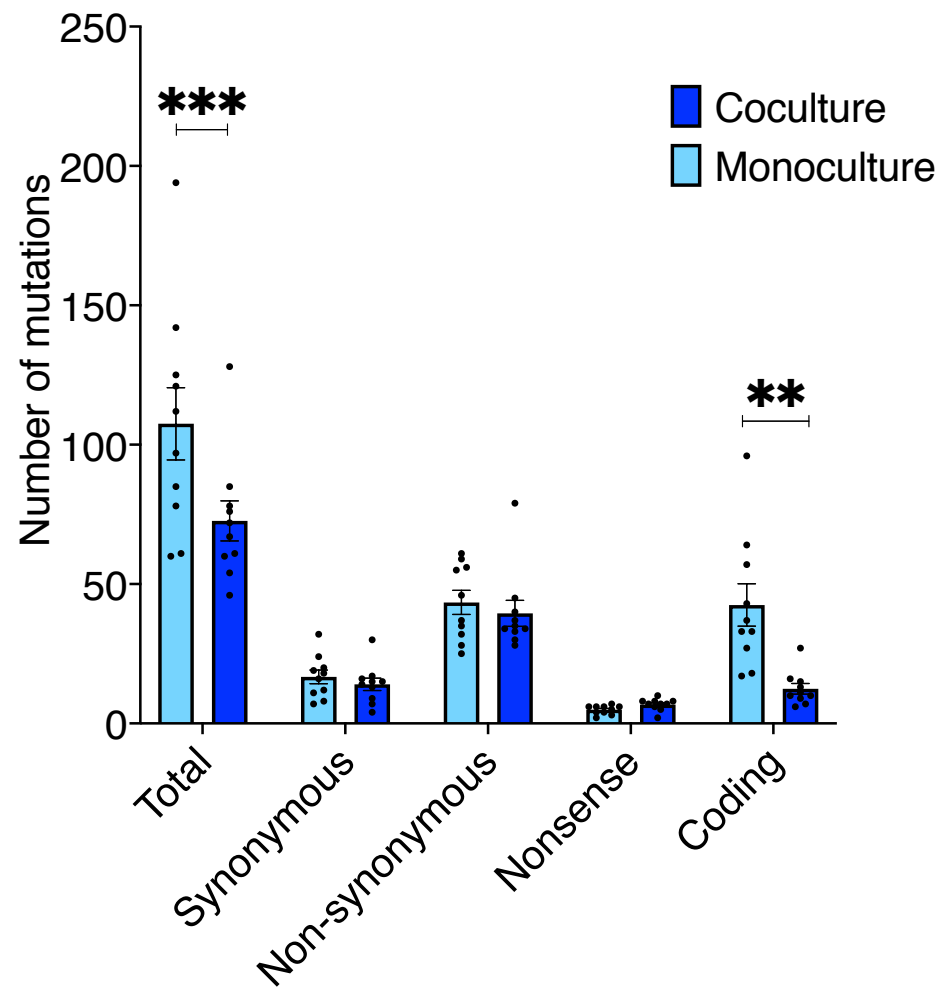

Supplement: Supplementary file 2 — Supplementary Figure 2. [file 41396_2022_1191_MOESM2_ESM.pdf]

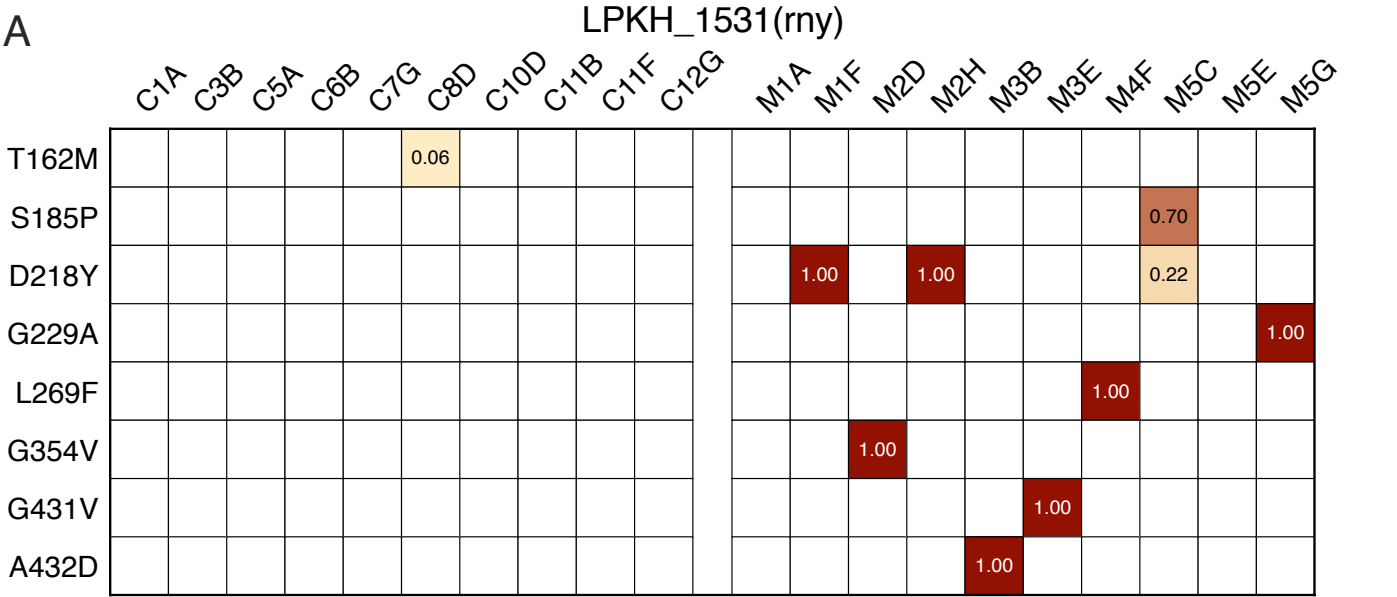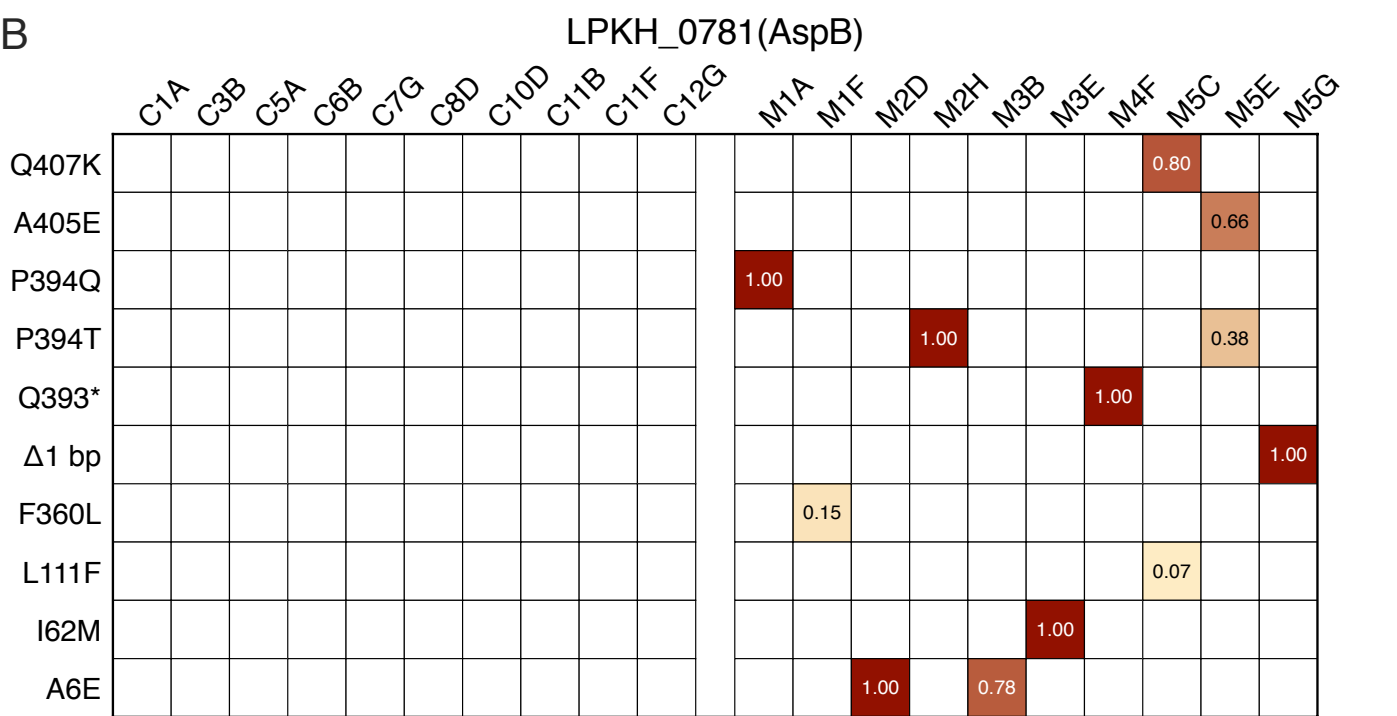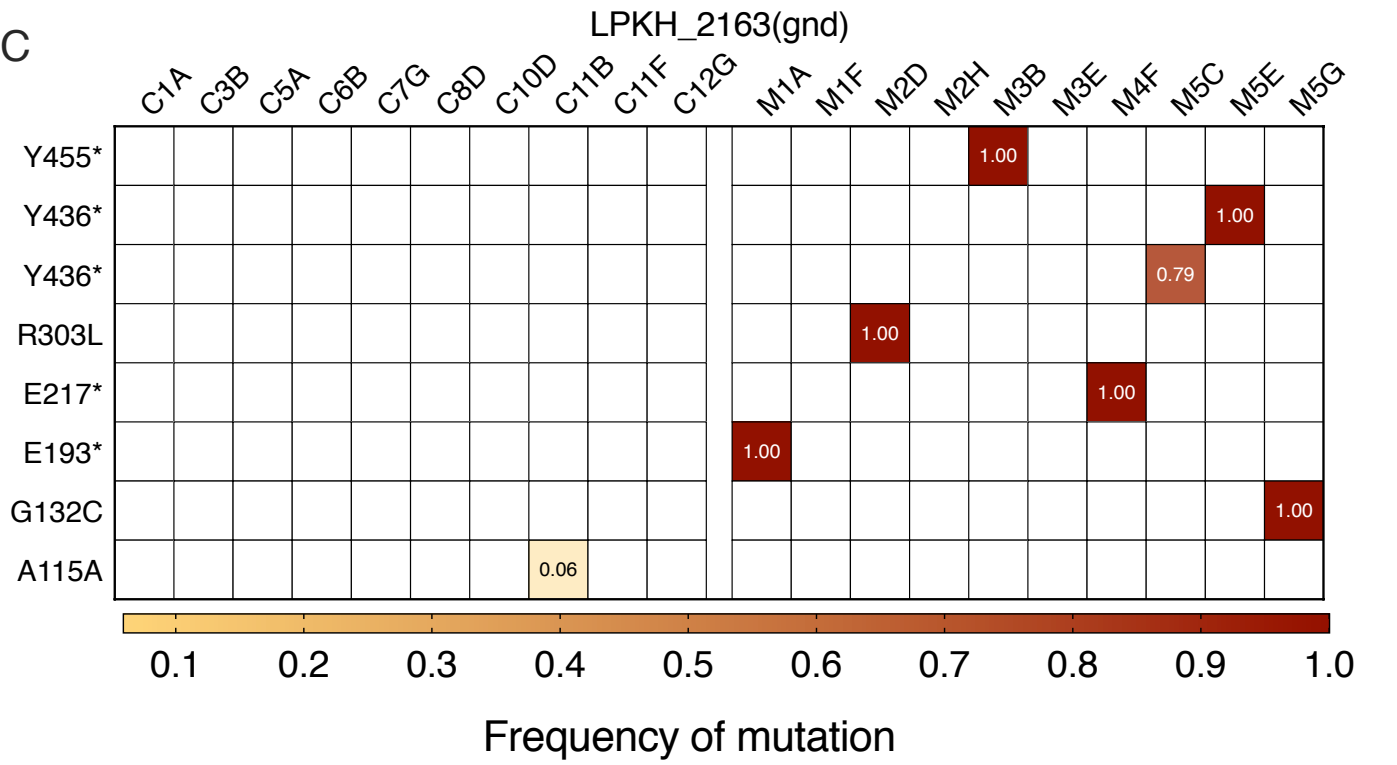

Supplement: Supplementary file 3 — Supplementary Figure 3 [file 41396_2022_1191_MOESM3_ESM.pdf]

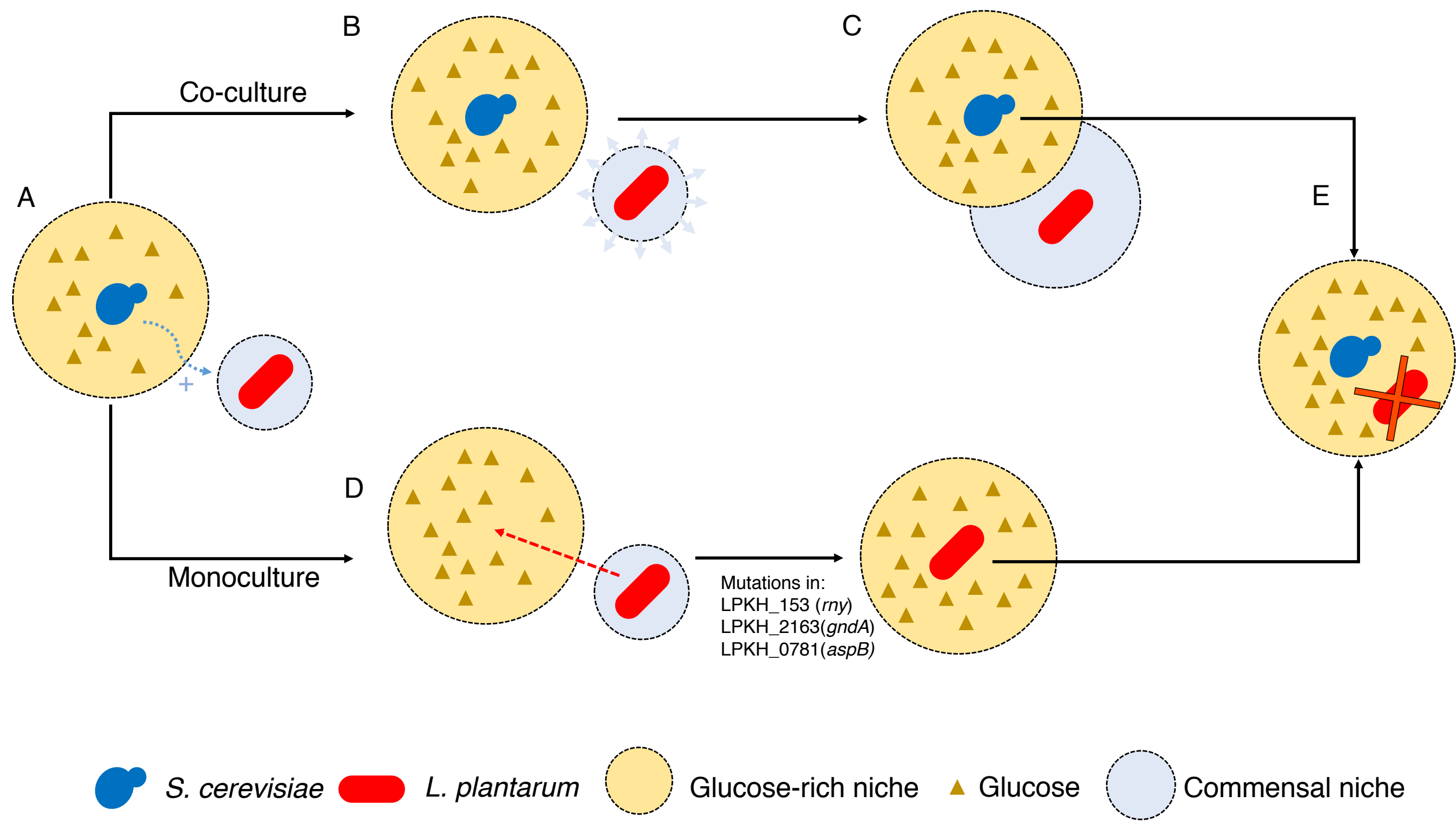

Supplement: Supplementary file 4 — Supplementary Figure 4 [file 41396_2022_1191_MOESM4_ESM.pdf]
