# Supplementary material for: Species interactions constrain adaptation and preserve ecological stability in an experimental microbial community
Source: ISME J. 2022 Jan 22;16(5):1442–52. doi: 10.1038/s41396-022-01191-1 (PMC9039033; doi:10.1038/s41396-022-01191-1)
Supplement: Supplementary file 5 — Supplementary Figure and Table Legends [file 41396_2022_1191_MOESM5_ESM.docx]

**Supplementary Figures**

**Supplementary Figure 1.**

**Supplementary Figure 1. Growth performance of *S. cerevisiae* and *L. plantarum* in CSM and the spent media of the other species.** Optical density (OD_600_) was tracked over time by destructive sampling of cultures at 3hr intervals (Methods). (A) *S. cerevisiae* and (B) *L. plantarum* in both complete supplement media (CSM) and the spent media of the other species. Error bars are the S.E.M.

**Supplementary Figure 2.**

**Supplementary Figure 2. Number and class of mutations in monoculture and co-culture evolved *L. plantarum* and *S. cerevisiae.*** Bars show the average number of mutations, and black dots show individual populations for whole genome sequenced *L. plantarum* **(A)** and *S. cerevisiae* **(B)**, (Methods). Error bars represent S.E.M.

**Supplementary Figure 3.**

**Supplementary Figure 3. Specific non-synonymous mutations in three highly parallel genes in monoculture *L. plantarum.*** Frequency of all mutations in sequenced co-culture and monoculture populations of *L. plantarum* within LPKH_1531(*rny*) **(A)**, LPKH_0781(*aspB*) **(B)**, and LPKH_2163(*gnd*) **(C)**. The specific amino acid change and the position is shown on the y-axis. Changes encoding stop codons denoted by *.

**Supplementary Figure 4.**

**Supplementary Figure 4. Schematic of evolutionary processes underlying coexistence dynamics in co-culture and monoculture *L. plantarum*.**

In the ancestral state of the pairwise community **(A)**, there is a commensal relationship between *S. cerevisiae* and *L. plantarum* (blue dotted arrow). The niche requirements of each strain are symbolised by the circles surrounding each strain, with *S. cerevisiae* occupying the “glucose niche” (yellow) and *L. plantarum* occupying the “commensal niche” (blue). As *L. plantarum* evolves in co-culture with *S. cerevisiae* **(B)**, evolution is constrained by *S. cerevisiae*, so that emerging ecotypes that would utilise glucose are selected against **(C)**, keeping niche requirements separate between the two species. In contrast, when *L. plantarum* is evolving in monoculture **(D)**, adaptation to the glucose rich niche is not selected against by interactions with *S. cerevisiae*, allowing *L. plantarum* ecological release to specialise on glucose as a carbon source (red arrow). Subsequently, when *L. plantarum* is reintroduced into co-culture **(E)**, *L. plantarum* is unable to survive with *S. cerevisiae* in the glucose rich niche, which leads to competitive exclusion of *L. plantarum*.

**Supplementary Table 1.**

Multihit genes in *S. cerevisiae*

*The maximum null value is the maximum expected number of hits in that gene in simulations of evolution without selection (Methods).

**Supplementary Table 2**

Multihit genes in *L. plantarum*
